# Differing dietary patterns according to body composition

**DOI:** 10.3389/fnut.2025.1509620

**Published:** 2025-07-22

**Authors:** Yea-Chan Lee, Hyung-Mi Kim, Hye Sun Lee, Soyoung Jeon, Yu-Jin Kwon, Ji-Won Lee

**Affiliations:** ^1^Department of Family Medicine, Severance Hospital, Yonsei University College of Medicine, Seoul, Republic of Korea; ^2^Department of Food and Nutrition, Dongduk Women’s University, Seoul, Republic of Korea; ^3^Biostatistics Collaboration Unit, Department of Research Affairs, Yonsei University College of Medicine, Seoul, Republic of Korea; ^4^Department of Family Medicine, Yongin Severance Hospital, Yonsei University College of Medicine, Seoul, Republic of Korea; ^5^Institute for Innovation in Digital Healthcare, Yonsei University, Seoul, Republic of Korea

**Keywords:** sarcopenia, obesity, sarcopenic obesity, body composition, nutrition

## Abstract

**Background:**

This study aimed to investigate dietary patterns in relation to body composition, categorized into four groups using large-scale nationwide data: neither low muscle mass nor high body fat (N), low muscle mass only (LMo), high body fat only (HFo), and low muscle mass with high body fat (LMHF).

**Methods:**

A total of 15,917 participants from the 2008–2011 Korea National Health and Nutrition Examination Survey were analyzed. Propensity score matching (PSM) values, representing the predicted probability of patients having LMo, HFo, or LMHF, were estimated using logistic regression analysis while adjusting for confounders. Analysis of covariance was then used to compare daily macronutrient intake and weekly consumption frequency of food groups among the study groups, adjusting for total calorie intake.

**Results:**

Participants in the LMHF and HFo groups consumed lower amounts of carbohydrates and higher proportions of proteins and fats compared to the N group. Those in the LMHF and LMo groups had less frequent consumption of rice and vegetables and more frequent intake of ultra-processed foods. Additionally, participants in the LMo and HFo groups consumed beverages more frequently than those in the N group.

**Conclusion:**

Imbalances in body composition, such as LMo, HFo, and LMHF, are associated with less favorable dietary patterns, including higher consumption of ultra-processed foods and beverages, and lower intake of rice and vegetables. Further research is needed to explore targeted nutritional interventions for these groups.

## 1 Introduction

Age-related changes in body composition, including an increase in fat mass and a substantial reduction in muscle mass, exert adverse effects on the physiologic function and metabolism of the human body (1). Many studies have noted that body fat distribution is closely related to metabolic dysfunction and disease, including impaired fasting glucose, insulin resistance, type 2 diabetes mellitus (DM), and cardiovascular diseases (CVD) (2–5). In a 2-year prospective cohort study, Son et al. suggested that low muscle mass is significantly associated with the incidence of type 2 DM, independent of obesity, in the Korean population (6). Furthermore, prospective studies have demonstrated associations between low muscle mass and an increased risk of CVD and all-cause mortality (7–10). Using dual-energy X-ray absorptiometry (DXA), Baumgartner first defined sarcopenic obesity as the co-presence of two conditions: (1) obesity, defined as percent body fat values above the median for each sex, and (2) sarcopenia, defined as an age-related change in muscle mass to height squared, with values falling two standard deviations below the mean of a healthy, younger population (11).

Sarcopenic obesity poses a critical risk to public health worldwide (12). Lim et al. reported that individuals with sarcopenic obesity with low appendicular skeletal muscle mass (ASM) and high visceral fat mass are at higher risk of metabolic syndrome and insulin resistance compared to those with either sarcopenia or obesity alone in the Korean population (13). Furthermore, a 10-year follow-up cohort study showed that participants with sarcopenic obesity carried a 23% higher risk of CVD, regardless of the lack of association between CVD risk and obesity or sarcopenia alone (14). The significant risks to public health outcomes posed by sarcopenic obesity emphasize the importance of preventive measures against imbalances in body composition (15).

Dietary patterns are a principal contributing factor to body composition, including sarcopenia and obesity (15, 16). Nutritional strategies have been designed in consideration of their effects on the development of sarcopenic obesity (15). Several studies have suggested that high protein intake combined with resistance exercise increases skeletal muscle mass and strength in an older adult population with low muscle mass (17, 18). According to a 13-week randomized controlled trial (RCT) in the Netherlands (2011–2012), a high intake of whey protein, leucine, and vitamin D supplements preserved skeletal muscle mass while prompting weight loss, in conjunction with resistance exercise three times a week, in older adults with high body fat (19). However, an RCT conducted in Denmark (2019–2021) found that, while a high-protein diet enhanced satiety, it did not significantly affect body composition or cardiometabolic markers in young women with overweight (20). These findings indicate that the effects of protein intake may vary depending on age, metabolic status, and exercise habits. Another area of controversy lies in the impact of macronutrient distribution on metabolic health. Parvaresh et al. reported that a high-carbohydrate diet has a distinct adverse effect on metabolic responses in obese individuals with insulin resistance, whereas high-protein or high-fat diets do not elicit such effects (21). On the other hand, a cross-sectional analysis from the 2011–2014 National Health and Nutrition Examination Survey (NHANES) found a positive association between dietary fat intake (both saturated and unsaturated fatty acids) and skeletal muscle mass, suggesting the potential need for adequate dietary fat intake to prevent sarcopenia (22). Meanwhile, a 13-week trial study conducted in the United States reported that dietary fat intake influenced intramyocellular lipid accumulation in both healthy and older individuals, which, in turn, negatively affected muscle metabolism, contributing to sarcopenia (23). Previous studies on sarcopenia and sarcopenic obesity have reported inconsistent findings, largely due to variations in study populations, methods used for assessing body composition and metabolism, and dietary intake measurement tools (22, 24). These discrepancies highlight the need for further research to clarify the relationship between dietary patterns and body composition characteristics.

Therefore, in this study, we aimed to examine whether individuals with low muscle mass only (LMo), high body fat only (HFo), or both conditions (LMHF) demonstrate distinct dietary behaviors compared to those with normal body composition. We hypothesized that these groups would differ in both macronutrient intake and the frequency of consuming specific food categories, reflecting variations in overall dietary quality. Using nationally representative data from the Korea National Health and Nutrition Examination Survey (KNHANES), we aimed to explore potential associations between diet and body composition in the Korean adult population.

## 2 Materials and methods

### 2.1 Study population

**Figure 1:**
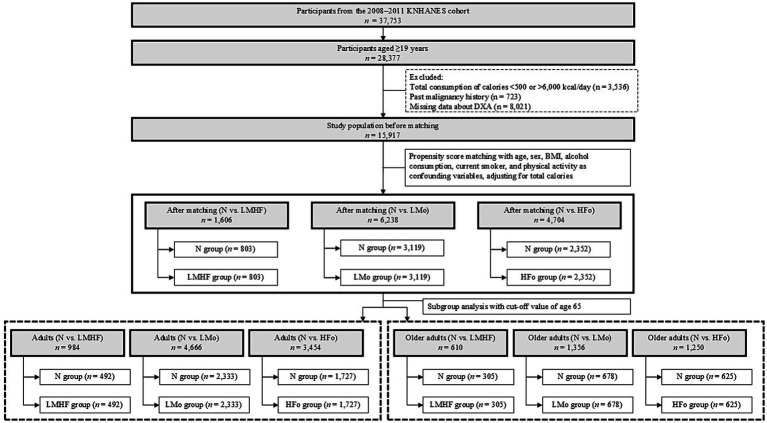
Flowchart of the study population selection. KNHANES, Korea National Health and Nutrition Examination Survey; DXA, dual-energy X-ray absorptiometry; BMI, body mass index; N, neither low muscle mass nor high body fat; LMHF, low muscle mass with high body fat; LMo, low muscle mass only, but not high body fat; HFo, high body fat only, but not low muscle mass.

We used data from the 2008–2011 KNHANES conducted by the Korea Centers for Disease Control and Prevention. Since 1998, KNHANES has been conducted to monitor health risk behaviors, including smoking, drinking, nutrition intake, physical activities, and major chronic diseases. This study included 28,377 participants aged ≥19 years. We excluded participants who consumed <500 kcal/day or >6,000 kcal/day (*n* = 3,536), had malignancy (*n* = 723), or had missing data from DXA (*n* = 8,021). After exclusion, 15,917 participants were included in the final analysis (Figure 1). Informed consent was obtained from all subjects before inclusion in the KNHANES, in accordance with the Declaration of Helsinki. The Institutional Review Board of Yongin Severance Hospital approved the study protocol (IRB No: 9–2019-0016).

### 2.2 Anthropometrics and biochemical variables

Body weight and height were measured to the nearest 0.1 kg and 0.1 cm, respectively. Body mass index (BMI) was calculated as weight in kilograms (kg) divided by the square of height in meters (m^2^). Waist circumference (WC) (cm) was measured as the circumference at the midpoint of a horizontal line between the superior margin of the iliac crest and the inferior margin of the twelfth rib. Systolic blood pressure (SBP) and diastolic blood pressure (DBP) were measured twice with the patient in a seated position after resting for at least 5 min, and the mean value of the two measurements was recorded. Blood samples were collected after a minimum of 8 h of fasting. Serum levels of triglyceride (TG), high-density lipoprotein cholesterol (HDL-C), and fasting plasma glucose (FPG) were analyzed using the Hitachi Automatic Analyzer 7,600 (Hitachi, Tokyo, Japan). Information on smoking, drinking, and exercise habits was obtained from self-reported questionnaires. Current alcohol drinkers were defined as those who consumed at least one glass of alcohol per week within the last year. A current smoker was defined as having ever smoked 100 or more cigarettes within one’s lifetime and smoking every day or some days at the time of survey (25). Physical activity was estimated as metabolic equivalent (MET)-min/week by multiplying the weekly spending time for an activity (min/week) by its intensity, which was four for mild to moderate intensity and eight for vigorous intensity, respectively (26, 27). Hypertension (HTN) was defined as SBP ≥ 140 mmHg, DBP ≥ 90 mmHg, or the use of antihypertensive medication. DM was defined as FPG ≥ 126 mg/dL or the use of anti-diabetic medication. Dyslipidemia was defined as a serum total cholesterol level ≥240 mg/dL or the use of antidyslipidemic medication.

### 2.3 Assessment of body composition

We utilized DXA (QDR 4500A; Hologic Inc., Bedford, MA, United States) to assess body composition. Fat mass was measured using a whole-body scan. ASM referred to the sum of lean muscle mass in both the arms and legs, and it was adjusted using height squared (ASM/height^2^). Low muscle mass was defined as a loss of muscle mass, indicated by an ASM/height^2^ < 7.0 kg/m^2^ in men and <5.4 kg/m^2^ in women, based on the 2019 Asian Working Group for Sarcopenia consensus (28). Moreover, the participants were considered as high body fat if they were in the highest quintile of the percentage of body fat mass measured by DXA (cutoff values of 39.2% for women and 27.3% for men). Along with the status of body composition, we divided the participants into four subgroups: the N group, participants with neither low muscle mass nor high body fat; the LMo group, participants with low muscle mass only, but not high body fat; the HFo group, participants with high body fat only, but not low muscle mass; and the LMHF group, participants with low muscle mass and high body fat concurrently.

### 2.4 Assessment of nutrition intake and dietary patterns

Face-to-face interviews were conducted by skilled dietitians to administer nutritional surveys. Food intake was examined using the 24-h recall method and semi-quantitative food frequency questionnaire (FFQ) (29). The 24-h recall method was used to estimate total energy and nutrient intake, while the FFQ, designed to reflect commonly consumed foods and key nutrient sources within the study population, assessed long-term dietary patterns. To minimize self-reporting errors and enhance standardization, the FFQ included 63 food items contributing to 90% of total energy and nutrient intake, with portion sizes standardized using two-dimensional models, measuring cups, and spoons. Detailed food items are described on the KNHANES website.^1^

### 2.5 Statistical analysis

Data from the KNHANES, which was designed as a complex survey, were used in this study. To ensure representativeness of the Korean population, sampling weights were applied during the analysis (30). Continuous variables were presented as mean values ± SD, and categorical variables were presented as numbers (percentage) in the data analysis. Analysis of variance (ANOVA) was used for analyzing continuous variables, whereas a chi-squared test was used for assessing categorical variables. Propensity score matching (PSM) values for the predicted probability of participants with LMo, HFo, or LMHF were estimated using logistic regression analysis, with age, sex, BMI, alcohol consumption, smoking status, and physical activity as confounding factors. The C-statistics of the logistic regression models were calculated to be 0.55 to 0.7. Considering the differences between the two groups, subjects without LMHF were matched to those in the LMHF group in a 1:1 ratio according to the nearest neighbor method with the greedy algorithm. Standardized differences were calculated to assess the balance of confounding factors after matching. All standardized differences were less than 0.2. Furthermore, 1:1 PS matching was applied to compare participants with and without LMo, as well as those with and without HFo. Subgroup analysis was performed based on age groups, classifying participants as either young to middle-aged adults (<65 years) or older adults (≥65 years). To compare the daily macronutrient intake and the weekly consumption frequency of food groups between groups, an analysis of covariance (ANCOVA) was used for continuous variables, after adjusting for total calories. ANCOVA was selected instead of simpler methods such as t-tests or ANOVA because the method incorporates covariate adjustment, specifically for total energy intake, thereby mitigating potential confounding and enhancing the precision of estimated intergroup differences in dietary patterns. The *post-hoc* power was calculated for the macronutrient variables, including carbohydrate, protein, and fat intake, and the power exceeding 0.8 confirms the sufficiency of the sample size to detect intergroup differences. *p*-values of <0.05 were considered statistically significant, and all statistical analyses were performed using SAS version 9.4 (SAS Institute, Cary, NC, United States).

## 3 Results

### 3.1 General and clinical characteristics of the study population

Table 1 presents the participants’ general characteristics according to the N, LMHF, LMo, and HFo groups. Compared to participants in the N group, those in the LMHF and HFo groups were significantly older and had higher WC, body fat, SBP, and FPG, whereas those in the LMo group were younger and had lower WC, body fat, SBP, and FPG. The mean BMI in the HFo group was higher than that in the N group (27.7 ± 2.9 kg/m^2^ vs. 23.8 ± 2.6 kg/m^2^), while these values in the LMHF and LMo groups were lower than those in the N group.

**Table 1:** General characteristics of the study population.

Variables	Total	N	LMHF	LMo	HFo
	*n* = 15,917	*n* = 9,582	*n* = 816	*n* = 3,152	*n* = 2,367
Age (years)	49.7 ± 16.4	49.1 ± 15.5	56.3 ± 17.2	48.3 ± 18.3	51.7 ± 16.3
Sex (% male)†	6,486 (40.8)	4,011 (41.9)	334 (40.9)	1,178 (37.4)	963 (40.7)
Body weight (kg)	61.9 ± 11.3	62.7 ± 10.0	59.6 ± 7.6	52.1 ± 6.9	72.1 ± 11.6
Height (cm)	161.7 ± 9.1	162.1 ± 9.2	159.5 ± 8.	161.3 ± 8.2	161.1 ± 9.8
BMI (kg/m^2^)	23.6 ± 3.3	23.8 ± 2.6	23.4 ± 1.8	20.0 ± 1.8	27.7 ± 2.9
WC (cm)	81.0 ± 10.0	81.2 ± 8.5	82.5 ± 7.9	72.2 ± 7.4	91.5 ± 8.5
ASM (kg)	17.5 ± 4.6	18.5 ± 4.6	14.4 ± 3.4	14.7 ± 3.1	18.4 ± 4.8
ASM/height^2^ (kg/m^2^)	6.6 ± 1.2	6.9 ± 1.1	5.6 ± 0.8	5.6 ± 0.8	7.0 ± 1.1
Body fat (kg)	16.6 ± 5.6	15.6 ± 4.5	20.2 ± 3.3	12.8 ± 3.7	24.5 ± 4.8
Body fat (%)	29.4 ± 8.3	27.5 ± 7.4	37.5 ± 6.3	27.4 ± 7.6	37.2 ± 6.3
SBP (mmHg)	119.6 ± 17.9	119.4 ± 17.4	123.0 ± 19.1	115.3 ± 18.2	125.0 ± 17.3
DBP (mmHg)	76.5 ± 10.8	76.7 ± 10.7	76.5 ± 10.5	73.4 ± 10.3	79.8 ± 10.5
Triglyceride (mg/dL)	132.1 ± 107.5	134.2 ± 112.9	137.9 ± 86.3	106.2 ± 85.7	156.2 ± 110.2
HDL-C (mg/dL)	52.5 ± 12.7	52.2 ± 12.6	51.3 ± 12.2	56.3 ± 13.2	48.9 ± 11.1
FPG (mg/dL)	97.6 ± 22.5	97.6 ± 22.2	100.3 ± 26.6	94.5 ± 22.8	101.1 ± 21.7
Current alcohol drinker†	3,415 (21.5)	2,199 (23.0)	113 (13.9)	571 (18.1)	532 (22.5)
Current smoker †	3,080 (19.4)	1939 (20.3)	111 (13.7)	631 (20.1)	399 (16.9)
Physical activity (MET-min/week)	1681.9 ± 3692.1	1939.2 ± 4005.7	1188.6 ± 2836.5	1198.7 ± 3236.5	1452.3 ± 3046.3
HTN†	5,081 (32.1)	2,914 (30.6)	369 (45.6)	681 (21.8)	1,117 (47.5)
DM†	1,445 (9.7)	871 (9.6)	111 (14.8)	203 (7.0)	260 (11.8)
Dyslipidemia†	1887 (12.7)	1,092 (12.0)	121 (16.2)	221 (7.6)	453 (20.6)

N, normal; LMHF, low muscle mass with high body fat; HFo, high body fat only, but not low muscle mass; LMo, low muscle mass only, but not high body fat; BMI, body mass index; WC, waist circumference; ASM, appendicular skeletal muscle mass; SBP, systolic blood pressure; DBP, diastolic blood pressure; HDL-cholesterol, high-density lipoprotein cholesterol; FPG, fasting plasma glucose; HTN, hypertension; DM, diabetes mellitus; ns, not significant. The values are presented as means ± standard deviations for continuous variables and numbers (%) for categorical variables. † Categorical variables.

The proportions of HTN, DM, and dyslipidemia were higher in the LMHF and HFo groups than in the N group, whereas they were lower in the LMo group than in the N group. Participants in the N group (1,939.2 ± 4,005.7 MET-min/week) engaged in more physical activity than those in any other group (1,188.6 ± 2,836.5, 1,198.7 ± 3,236.5, and 1,452.3 ± 3,046.3 MET-min/week in the LMHF, LMo, and HFo groups, respectively).

The mean age of the HFo group was lower than that of the LMHF group (51.7 ± 16.3 vs. 56.3 ± 17.2). Additionally, the mean values of body weight, height, BMI, WC, ASM, ASM/height^2^, body fat, SBP, DBP, TG, and HDL-C were higher in the HFo group than in the LMHF group. Participants in the LMo group had lower BMI, lower proportion of body fat mass, lower TG levels, and higher HDL-C levels compared to those in the LMHF group. The proportions of current alcohol drinkers and smokers were higher in the LMo and HFo groups than in the LMHF group. Moreover, a lower proportion of DM was observed in the LMo (7.0%) and HFo (11.8%) groups than in the LMHF group (14.8%). Additionally, the proportion of dyslipidemia was lower in the LMo group (7.6%) and higher in the HFo group (20.6%) compared to the LMHF group (16.2%).

### 3.2 Comparison of daily macronutrient intake and weekly food consumption frequency after propensity score matching analysis

**Figure 2:**
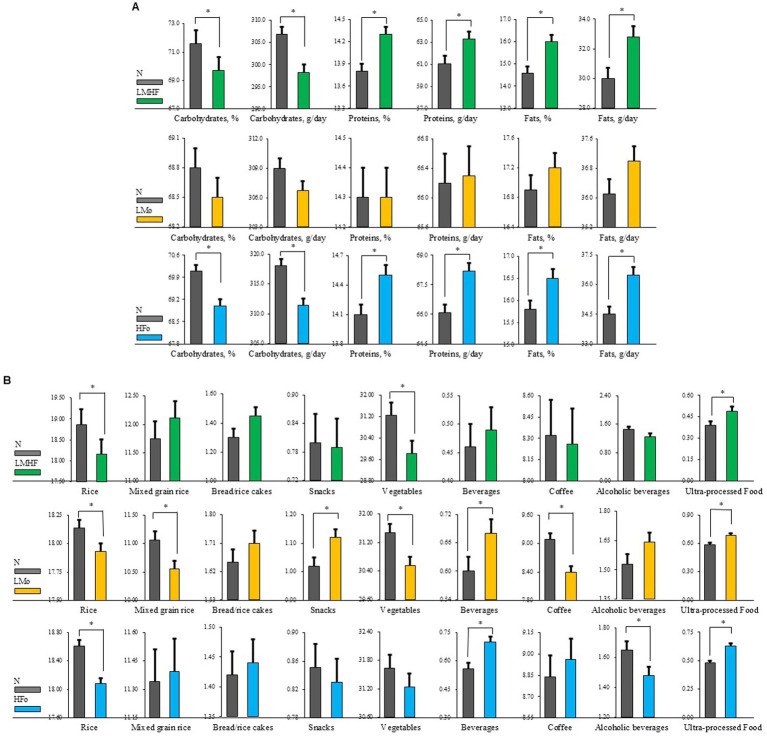
Comparison of nutrient intake and dietary patterns after propensity score matching analysis between the two groups, adjusting for total calorie intake. **(A)** Comparison of daily intake of macronutrients (carbohydrate, protein, and fat). **(B)** Comparison of weekly consumption frequency (times per week) of various food groups, including traditional staples (e.g., rice, mixed grain rice, and vegetables) and items typically considered unhealthy (e.g., snacks, beverages, and ultra-processed foods). Higher consumption of ultra-processed foods and lower intake of rice and vegetables are dietary patterns often associated with poor diet quality. These observed trends provide visual support for the dietary imbalances discussed in the main text. N, neither low muscle mass nor high body fat; LMHF, low muscle mass with high body fat; LMo, low muscle mass only, but not high body fat; HFo, high body fat only, but not low muscle mass. *p-value of less than 0.05.

In total, 803 pairs in the N vs. LMHF comparison, 3,119 pairs in the N vs. LMo comparison, and 2,352 pairs in the N vs. HFo comparison were matched in the PS matching analyses. Daily macronutrient intake values for the matched sets are shown in Figure 2A and Appendix Table A1. Daily Carbohydrate (CHO) intake (g) and the proportion of CHO intake (%) were significantly lower, whereas daily protein and fat intake and the proportion of protein and fat intake were higher in the LMHF group than in the N group. There was no significant difference in macronutrient intake between the N and LMo groups. Compared with the participants in the N group, those in the HFo group consumed fewer CHOs, more proteins, and more fats daily.

Weekly food consumption frequencies for the matched sets are shown in Figure 2B and Appendix Table A1. There were significant differences in the weekly consumption of food groups between the LMHF and N groups, including the consumption of ultra-processed food, particularly hamburger, which was more frequent in the LMHF group than in the N group [mean (SE): 0.49 (0.03) vs. 0.39 (0.03), *p*-value = 0.025]. Participants in the LMHF group consumed rice and vegetables less frequently than those in the N group. Moreover, individuals in the LMo [mean (SE): 0.69 (0.02) vs. 0.59 (0.02), *p*-value <0.001] and HFo groups [0.63 (0.02) vs. 0.48 (0.02), *p*-value <0.001] showed significantly higher intake frequencies of ultra-processed foods, including hamburger, pizza, and fried foods, compared to the N group. Participants in the LMo group also showed higher frequencies of snack and beverage consumption, while consuming rice, mixed grain rice, vegetables, and coffee less frequently than participants in the N group. Weekly consumption frequencies for the HFo group revealed higher consumption of beverages and lower consumption of rice compared to the N group.

### 3.3 Subgroup analyses of young to middle-aged adults and older adults

**Figure 3:**
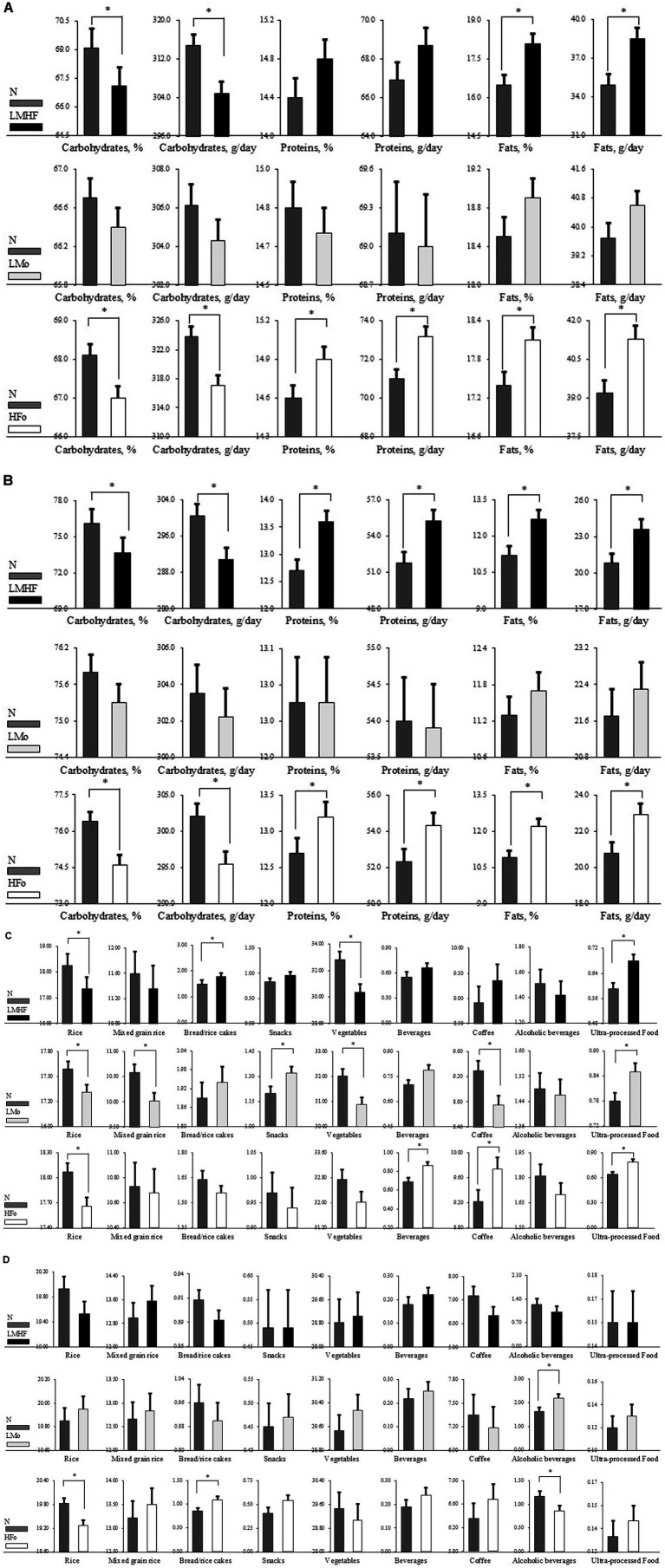
Comparison of nutrient intake and dietary patterns between young to middle-aged adults and older adults. **(A)** Comparison of daily nutrient intake in young to middle-aged adults. **(B)** Comparison of daily nutrient intake in older adults. **(C)** Comparison of weekly consumption frequency (times per week) of various food groups in young to middle-aged adults. **(D)** Comparison of weekly food consumption frequency in older adults. N, neither low muscle mass nor high body fat; LMHF, low muscle mass with high body fat; LMo, low muscle mass only, but not high body fat; HFo, high body fat only, but not low muscle mass. *p-value of less than 0.05.

In the subgroup analyses of young to middle-aged adults (aged <65 years), 984 pairs were matched in the N vs. LMHF comparison, 4,666 pairs in the N vs. LMo comparison, and 3,454 pairs in the N vs. HFo comparison. In the subgroup analyses of older adults (aged >65 years), 610 pairs were matched in the N vs. LMHF comparison, 1,356 pairs in the N vs. LMo comparison, and 1,250 pairs in the N vs. HFo comparison. Daily macronutrient intake values for the matched sets of young to middle-aged adults and older adults are shown in Figures 3A,B, respectively. Appendix Table A2 demonstrates the values of daily macronutrient intake in the matched sets of both subgroups. Among older adults, the LMHF group showed lower intake of CHOs [mean (SE): 290.9 (2.5) vs. 300.5 (2.5) g/day, *p*-value <0.05] and higher consumption of fats [23.6 (0.8) vs. 20.8 (0.8) g/day, *p*-value <0.05] compared to the N group.

Figures 3C,D, and Appendix Table A2 show the weekly consumption frequencies of food groups for the matched sets of young to middle-aged adults and older adults. After 1:1 PS matching of young to middle-aged adults, individuals in the LMHF, LMo, and HFo groups consumed ultra-processed food more frequently compared to those in the N group. Similar to the findings from the PS matching analysis of the entire cohort (Figure 2B), participants in the young to middle-aged adult LMHF group showed significantly less weekly consumption of rice [mean (SE): 17.34 (0.19) vs. 18.25 (0.19)] and vegetables [30.36 (0.63) vs. 32.82 (0.62)] than those in the young to middle-aged adult N group. Furthermore, the weekly consumption of vegetables in the LMo group of young to middle-aged adults was significantly lower than that in the N group [mean (SE): 30.88 (0.28) vs. 32.01 (0.28)]. In addition, participants in the young to middle-aged adult HFo group consumed rice less frequently and beverages and ultra-processed food more frequently than those in the young to middle-aged adult N group. Older adults in the HFo group consumed rice less frequently than those in the N group. No significant differences were observed in weekly food consumption frequencies among older adults when comparing the LMHF vs. N and LMo vs. N groups.

## 4 Discussion

In this study, we found that participants in the LMHF and HFo groups consumed less CHOs and higher proportions of proteins and fats compared to the N group. However, despite this higher protein intake, these groups still exhibited adverse body composition outcomes, contradicting the widely held assumption that protein intake protects against sarcopenic obesity. Notably, those in the LMHF and LMo groups reported less frequent consumption of rice and vegetables and more frequent intake of ultra-processed foods, a trend that aligns with the increasing prevalence of Westernized dietary patterns in the Republic of Korea. Additionally, participants in the LMo and HFo groups consumed beverages more frequently than those in the N group, further indicating an increased reliance on sugar-sweetened drinks and processed food sources. While carbohydrate restriction is often suggested to improve metabolic health, our findings suggest that imbalanced macronutrient intake, particularly in the form of ultra-processed foods and excessive dietary fats, may contribute to poor body composition outcomes despite higher protein intake.

The shift from a traditional Korean diet to a Westernized dietary pattern is reflected in the LMHF and HFo groups, which showed increased ultra-processed food and beverage consumption and reduced intake of traditional staples such as rice and vegetables. Traditionally, Korean-style balanced diets, which emphasize grains, vegetables, and fermented foods, have been associated with a lower risk of metabolic disorders (31, 32). However, recent trends indicate a decline in rice and vegetable consumption and an increase in processed food intake, paralleling the increase in obesity and metabolic syndrome in the Republic of Korea (33, 34). Our findings align with this dietary transition, as participants in the LMHF and HFo groups consumed fewer carbohydrates, more proteins and fats, and a higher proportion of ultra-processed foods, especially hamburger, compared to the N group. Similarly, participants in the LMo group also exhibited significantly lower consumption of rice and vegetables while relying more on beverages and processed foods. These patterns suggest that dietary shifts toward a Westernized eating pattern may contribute to unhealthy body composition (32), particularly in groups with imbalanced macronutrient intake.

While previous research has emphasized the benefits of carbohydrate restriction and adequate protein intake in managing sarcopenic obesity (15, 35–37), these studies often overlooked the role of overall dietary quality. Our findings fill this gap by demonstrating that participants in the LMHF group—despite lower carbohydrate and higher protein consumption—still exhibited unfavorable body composition. This paradox suggests that nutrient composition alone is insufficient; instead, dietary quality and food source are crucial determinants. Increased intake of ultra-processed foods, rich in sugar, salt, and saturated fats, may undermine the benefits of higher protein consumption by promoting inflammation and impairing muscle protein synthesis (38). Furthermore, protein efficacy may vary depending on its source, with high-quality proteins such as whey or soy offering greater benefits than processed or animal-based alternatives (39). These insights underscore the importance of moving beyond macronutrient-focused approaches toward holistic dietary assessments when addressing sarcopenic obesity.

Our findings are consistent with global studies, such as a French cohort study (2009–2019) that reported a positive correlation between ultra-processed food consumption and obesity (40) and a UK Biobank study that demonstrated a link between ultra-processed food intake and increased body fat (41).

One possible explanation for this phenomenon lies in the source and quality of protein intake. Although previous studies supported protein’s role in preserving lean mass (42–44), emerging evidence suggests that plant-based or minimally processed proteins may have more favorable effects on metabolic health compared to animal-based or highly processed sources (45). A more detailed analysis distinguishing between protein sources or categorizing fats into saturated and unsaturated types could yield deeper insights. In addition, the role of specific food components in metabolic regulation warrants attention. A 3-week intervention study involving polyphenol-rich pomegranate juice and fermented soy showed improvements in plasma antioxidant capacity and a reduction in lipid peroxidation, as evidenced by lower levels of thiobarbituric acid reactive substances (46). Beyond physiological biomarkers, a follow-up nutrigenomic analysis from the same study revealed subtle but meaningful changes in miRNA expression related to oxidative stress and immune modulation, as detectable through melt curve profiling (47). These findings underscore the importance of considering not only dietary content but also the molecular responses it elicits when evaluating dietary interventions aimed at improving body composition and metabolic health.

The lower carbohydrate intake observed in the LMHF and HFo groups may reflect a broader shift toward Westernized dietary patterns, which are typically high in ultra-processed foods and refined fats and low in dietary fiber. These patterns not only lower dietary quality but also impact gut microbial composition, a key mediator of metabolic health (32). Unlike traditional Korean diets rich in plant-based foods and fermented products, Western-style diets have been associated with decreased microbial diversity and increased abundance of taxa such as Bacteroides, which are linked to proteolytic activity and reduced fermentation of complex carbohydrates. This microbial shift may lead to increased gut permeability and low-grade inflammation, contributing to unfavorable body composition, including visceral fat accumulation and muscle loss (32). Supporting this shift, De Filippo et al. demonstrated that children consuming Western diets showed greater proteolytic fermentation and reduced microbiota richness compared to those following traditional, fiber-rich diets. These findings illustrate how habitual dietary intake influences gut ecology, which in turn may affect nutrient utilization and host metabolism (48). Taken together, this suggests that dietary patterns shape not only macronutrient balance but also microbiome-mediated mechanisms that may influence the development of sarcopenic obesity.

Frequent beverage consumption, particularly sugary drinks, has also been linked to adverse body composition outcomes. A 6-month randomized intervention study in 2012 by Maria et al. reported that beverages, including soft drinks, sugar-sweetened tea, and coffee with cream and artificial sweeteners, are associated with ectopic fat accumulation and increased visceral fat (49). Other studies have found that fructose from sugar-sweetened beverages promotes *de novo* lipogenesis in the liver, leading to fat accumulation in both skeletal muscle and visceral adipose tissue (50–53). These mechanisms suggest that beverage consumption may contribute to the development of body composition imbalances, including low muscle mass and high body fat.

Our study had several limitations. First, as a cross-sectional study, it identifies associations between body composition and dietary patterns but cannot establish causality. A deeper exploration of how specific dietary components directly influence muscle mass and fat accumulation is needed. Second, although we applied propensity score matching (PSM) to reduce confounding, unmeasured variables such as meal timing, supplement use, or chronic inflammation status were not accounted for. These factors may influence both dietary behavior and body composition, leading to residual confounding. The PSM technique, while valuable for improving group comparability, may have excluded participants with atypical dietary patterns or rare phenotypes. This technique could reduce the diversity of the sample and potentially limit the external validity of our findings when generalizing to the broader population. Third, the nutritional data obtained from the 24-h recall method and FFQ may be subject to inaccuracies due to reliance on dietary recall rather than precise nutrient recording. Fourth, a sub-analysis distinguishing protein sources (animal vs. plant) and fat types (saturated vs. unsaturated) could have provided deeper insights into body composition imbalances; however, our dataset’s limitations precluded such analyses. Fifth, while our study utilized large-scale national data, the final sample size may have been insufficient to fully capture population-level trends. Future research with larger cohorts and more detailed dietary assessments is needed to further elucidate these associations.

Despite these limitations, our investigation possesses several notable strengths. To our knowledge, this is the first nationwide study to compare dietary behaviors among individuals with LMo, HFo, and both conditions (LMHF) in a Korean adult population. Unlike previous studies focusing on single outcomes or limited age groups, our study uses a large, representative dataset (KNHANES) and robust PSM to provide an integrated analysis across distinct body composition phenotypes. Our findings also reflect Korea’s dietary transition—declining rice and vegetable intake and rising ultra-processed food consumption, which may contribute to the increasing prevalence of sarcopenic obesity. By identifying phenotype-specific dietary patterns, this study offers direction for tailored nutritional interventions.

## 5 Conclusion

Taken together, our study provides compelling evidence that dietary quality—not just nutrient quantity—is a critical determinant of body composition. This study fills an important gap in the literature by highlighting how modern dietary transitions in the Republic of Korea, such as increased ultra-processed food consumption, may contribute to the dual burden of obesity and sarcopenia. These insights emphasize the need to move beyond nutrient-specific guidelines and develop comprehensive, personalized dietary strategies for clinical practice and public health policy. Given the increasing prevalence of obesity and sarcopenic obesity in the Republic of Korea, further research is warranted to elucidate the underlying mechanisms of these associations and to assess the long-term metabolic consequences of evolving dietary patterns.

## Data Availability

The original contributions presented in the study are included in the article and Supplementary material. Further inquiries can be directed to the corresponding authors.

## Footnotes

^1^ https://knhanes.kdca.go.kr/knhanes/main.do
