# Supplementary material for: Differing dietary patterns according to body composition
Source: Front Nutr. 2025 Jul 22;12:1509620. doi: 10.3389/fnut.2025.1509620 (PMC12321545; doi:10.3389/fnut.2025.1509620)
Supplement: Supplementary file 1 [file Table_1.DOCX]

**Appendix A**

**Table A1.** Daily macronutrient intake and weekly consumption frequencies (times per week) of food groups according to body composition after propensity score matching analysis between two groups.

| **Variables** | **Normal**  ***n* = 803** | **LMHF**  ***n* = 803** | **P-value** | **Effect size** | **Normal**  ***n* = 3,119** | **LMo**  ***n* = 3,119** | **P-value** | **Effect size** | **Normal**  ***n* = 2,352** | **HFo**  ***n* = 2,352** | **P-value** | **Effect size** |
| --- | --- | --- | --- | --- | --- | --- | --- | --- | --- | --- | --- | --- |
| Carbohydrates (%) | 71.6  (70.8-72.3) | 69.7  (68.9-70.4) | <0.001 | 0.091 | 68.8  (68.4-69.1) | 68.5  (68.1-68.8) | 0.253 | 0.014 | 70.1  (69.7-70.6) | 69.0  (68.6-69.4) | <0.001 | 0.055 |
| Carbohydrates (g) | 306.8  (303.2-310.4) | 298.3  (294.7-301.8) | 0.001 | 0.082 | 309.0  (307.1-310.8) | 306.7  (304.8-308.5) | 0.090 | 0.021 | 318.1  (315.9-320.3) | 311.4  (309.2-313.6) | <0.001 | 0.061 |
| Proteins (%) | 13.8  (13.5-14.1) | 14.3  (14.0-14.6) | 0.012 | 0.063 | 14.3  (14.2-14.5) | 14.3  (14.2-14.5) | 0.903 | 0.002 | 14.1  (14.0-14.3) | 14.5  (14.3-14.6) | 0.003 | 0.043 |
| Proteins (g) | 61.1  (59.8-62.4) | 63.3  (61.9-64.6) | 0.024 | 0.056 | 66.2  (65.5-66.8) | 66.3  (65.6-67.0) | 0.741 | 0.004 | 66.1  (65.2-66.9) | 68.2  (67.4-69.1) | <0.001 | 0.051 |
| Fats (%) | 14.6  (14.1-15.2) | 16.0  (15.5-16.6) | <0.001 | 0.084 | 16.9  (16.6-17.2) | 17.2  (16.9-17.5) | 0.172 | 0.017 | 15.8  (15.4-16.1) | 16.5  (16.2-16.9) | <0.001 | 0.048 |
| Fats (g) | 30.0  (28.7-31.3) | 32.8  (31.5-34.1) | 0.003 | 0.075 | 36.1  (35.4-36.7) | 37.0  (36.3-37.7) | 0.055 | 0.024 | 34.5  (33.7-35.3) | 36.5  (35.7-37.3) | <0.001 | 0.052 |
| Weekly consumption frequency (times per week) of food groups | | | | | | | | | | | | |
| Rice | 18.87 (18.60-19.15) | 18.15 (17.87-18.42) | <0.001 | 0.092 | 18.14 (18.00-18.29) | 17.93 (17.78-18.07) | 0.042 | 0.026 | 18.61 (18.45-18.77) | 18.08 (17.92-18.24) | <0.001 | 0.068 |
| Mixed grain rice | 11.75 (11.16-12.34) | 12.11 (11.52-12.70) | 0.396 | 0.022 | 11.06 (10.77-11.35) | 10.55 (10.26-10.85) | 0.017 | 0.031 | 11.34 (11.00-11.68) | 11.40 (11.06-11.74) | 0.800 | 0.004 |
| Bread and rice cakes | 1.30 (1.18-1.43) | 1.45 (1.33-1.58) | 0.101 | 0.042 | 1.65 (1.58-1.73) | 1.71 (1.63-1.78) | 0.304 | 0.013 | 1.42 (1.34-1.49) | 1.44 (1.37-1.51) | 0.682 | 0.006 |
| Snacks | 0.80 (0.69-0.91) | 0.79 (0.68-0.90) | 0.911 | 0.003 | 1.02 (0.95-1.08) | 1.14 (1.08-1.21) | 0.008 | 0.034 | 0.85 (0.79-0.92) | 0.83 (0.77-0.90) | 0.686 | 0.006 |
| Vegetables | 31.25 (30.33-32.16) | 29.83 (28.91-30.75) | 0.033 | 0.054 | 31.48 (31.00-31.96) | 30.55 (30.07-31.03) | 0.007 | 0.034 | 31.63 (31.08-32.19) | 31.24 (30.69-31.80) | 0.326 | 0.014 |
| Beverages | 0.46 (0.38-0.55) | 0.49 (0.41-0.58) | 0.597 | 0.013 | 0.60 (0.55-0.65) | 0.68 (0.62-0.73) | 0.039 | 0.027 | 0.56 (0.51-0.62) | 0.70 (0.64-0.76) | 0.001 | 0.048 |
| Coffee | 8.32 (7.84-8.80) | 8.26 (7.77-8.74) | 0.858 | 0.005 | 9.09 (8.83-9.34) | 8.39 (8.13-8.64) | <0.001 | 0.049 | 8.84 (8.55-9.13) | 8.96 (8.66-9.25) | 0.589 | 0.008 |
| Alcoholic beverages | 1.45 (1.26-1.63) | 1.25 (1.06-1.44) | 0.138 | 0.038 | 1.53 (1.42-1.63) | 1.64 (1.54-1.75) | 0.119 | 0.020 | 1.65 (1.54-1.76) | 1.48 (1.37-1.59) | 0.039 | 0.030 |
| Ultra-processed food | 0.39 (0.34-0.45) | 0.49 (0.43-0.54) | 0.025 | 0.057 | 0.59 (0.56-0.63) | 0.69 (0.65-0.72) | <0.001 | 0.046 | 0.48 (0.44-0.52) | 0.63 (0.59-0.66) | <0.001 | 0.074 |
| Hamburger | 0.05 (0.03-0.07) | 0.08 (0.06-0.10) | 0.010 | 0.065 | 0.08 (0.07-0.10) | 0.12 (0.11-0.13) | <0.001 | 0.056 | 0.06 (0.05-0.07) | 0.09 (0.08-0.10) | 0.001 | 0.050 |
| Pizza | 0.08 (0.07-0.10) | 0.09 (0.07-0.10) | 0.521 | 0.016 | 0.11 (0.11-0.12) | 0.13 (0.12-0.14) | 0.004 | 0.036 | 0.09 (0.08-0.10) | 0.11 (0.10-0.12) | 0.001 | 0.049 |
| Fried food | 0.26 (0.22-0.30) | 0.32 (0.28-0.36) | 0.065 | 0.047 | 0.39 (0.37-0.42) | 0.43 (0.41-0.46) | 0.030 | 0.028 | 0.34 (0.31-0.36) | 0.43 (0.40-0.46) | <0.001 | 0.066 |

LMHF, low muscle mass with high body fat; HFo, high body fat only, but not low muscle mass; LMo, low muscle mass only, but not high body fat. Ultra-processed food includes hamburger, pizza, and fried food, which were also analyzed separately. The values are presented as ‘estimated mean (95% confidence interval)’ for each variable. *P*- values are calculated using the analysis of covariance after adjusting for total calories.

**Table A2.** Daily macronutrient intake and weekly consumption frequencies (times per week) of food groups according to body composition after propensity score matching analysis between two groups in young to middle-aged adults and older adults.

| **Young to middle-aged adults (<65 years)** | | | | | | | | | | | | |
| --- | --- | --- | --- | --- | --- | --- | --- | --- | --- | --- | --- | --- |
| **Variables** | **Normal**  ***n* = 492** | **LMHF**  ***n* = 492** | **P-value** | **Effect size** | **Normal**  ***n* = 2,333** | **LMo**  ***n* = 2,333** | **P-value** | **Effect size** | **Normal**  ***n* = 1,727** | **HFo**  ***n* = 1,727** | **P-value** | **Effect size** |
| Carbohydrates (%) | 69.1  (68.1-70.0) | 67.1  (66.2-68.0) | 0.003 | 0.095 | 66.7  (66.3-67.1) | 66.4  (66.0-66.8) | 0.314 | 0.015 | 68.1  (67.6-68.6) | 67.0  (66.5-67.5) | 0.002 | 0.053 |
| Carbohydrates (g) | 314.7  (310.0-319.3) | 304.8  (300.2-309.4) | 0.003 | 0.094 | 306.1  (303.9-308.3) | 304.3  (302.1-306.5) | 0.248 | 0.017 | 323.8  (321.2-326.5) | 317.1  (314.4-319.7) | <0.001 | 0.060 |
| Proteins (%) | 14.4  (14.1-14.8) | 14.8  (14.4-15.1) | 0.201 | 0.041 | 14.8  (14.6-15.0) | 14.7  (14.6-14.9) | 0.701 | 0.006 | 14.6  (14.4-14.8) | 14.9  (14.7-15.1) | 0.014 | 0.042 |
| Proteins (g) | 66.9  (65.1-68.6) | 68.7  (66.9-70.4) | 0.163 | 0.045 | 69.1  (68.2-70.0) | 69.0  (68.1-69.8) | 0.816 | 0.003 | 71.0  (70.0-72.0) | 73.2  (72.1-74.2) | 0.004 | 0.049 |
| Fats (%) | 16.5  (15.8-17.3) | 18.1  (17.4-18.9) | 0.003 | 0.096 | 18.5  (18.2-18.8) | 18.9  (18.5-19.2) | 0.154 | 0.021 | 17.4  (17.0-17.7) | 18.1  (17.7-18.5) | 0.008 | 0.045 |
| Fats (g) | 34.9  (33.2-36.6) | 38.5  (36.8-40.2) | 0.004 | 0.091 | 39.7  (38.9-40.5) | 40.6  (39.8-41.3) | 0.128 | 0.022 | 39.2  (38.2-40.2) | 41.3  (40.3-42.2) | 0.003 | 0.051 |
| Weekly consumption frequency (times per week) of food groups | | | | | | | | | | | | |
| Rice | 18.25 (17.89-18.62) | 17.34 (16.98-17.71) | 0.001 | 0.111 | 17.59 (17.42-17.77) | 17.31 (17.13-17.48) | 0.022 | 0.034 | 18.07 (17.88-18.27) | 17.66 (17.47-17.86) | 0.004 | 0.050 |
| Mixed grain rice | 11.59 (10.88-12.31) | 11.35 (10.63-12.07) | 0.643 | 0.015 | 10.59 (10.26-10.91) | 10.02 (9.70-10.35) | 0.016 | 0.036 | 10.73 (10.35-11.10) | 10.68 (10.30-11.06) | 0.869 | 0.003 |
| Bread and rice cakes | 1.49 (1.32-1.66) | 1.78 (1.61-1.96) | 0.017 | 0.077 | 1.89 (1.80-1.97) | 1.94 (1.85-2.02) | 0.431 | 0.012 | 1.64 (1.55-1.73) | 1.56 (1.47-1.65) | 0.224 | 0.021 |
| Snacks | 0.83 (0.69-0.97) | 0.96 (0.82-1.10) | 0.220 | 0.040 | 1.20 (1.12-1.28) | 1.32 (1.24-1.40) | 0.039 | 0.030 | 0.97 (0.90-1.05) | 0.94 (0.86-1.01) | 0.540 | 0.011 |
| Vegetables | 32.82 (31.60-34.05) | 30.36 (29.13-31.59) | 0.006 | 0.089 | 32.01 (31.46-32.56) | 30.88 (30.33-31.43) | 0.004 | 0.042 | 32.73 (32.08-33.37) | 32.01 (31.36-32.66) | 0.123 | 0.026 |
| Beverages | 0.55 (0.44-0.67) | 0.66 (0.54-0.77) | 0.219 | 0.040 | 0.70 (0.64-0.77) | 0.79 (0.73-0.85) | 0.220 | 0.027 | 0.69 (0.62-0.77) | 0.86 (0.79-0.94) | 0.001 | 0.055 |
| Coffee | 8.92 (8.30-9.54) | 9.36 (8.74-9.99) | 0.325 | 0.032 | 9.30 (9.01-9.60) | 8.75 (8.45-9.05) | 0.009 | 0.038 | 9.22 (8.87-9.57) | 9.74 (9.39-10.09) | 0.037 | 0.036 |
| Alcoholic beverages | 1.51 (1.29-1.72) | 1.42 (1.20-1.64) | 0.578 | 0.018 | 1.48 (1.38-1.59) | 1.46 (1.36-1.57) | 0.796 | 0.004 | 1.81 (1.68-1.94) | 1.70 (1.57-1.83) | 0.276 | 0.019 |
| Ultra-processed food | 0.59 (0.56-0.62) | 0.68 (0.64-0.73) | 0.016 | 0.045 | 0.78 (0.76-0.81) | 0.85 (0.83-0.88) | 0.007 | 0.027 | 0.64 (0.59-0.69) | 0.79 (0.74-0.84) | <0.001 | 0.071 |
| Hamburger | 0.08 (0.05-0.10) | 0.12 (0.09-0.14) | 0.026 | 0.072 | 0.12 (0.11-0.14) | 0.15 (0.14-0.17) | 0.005 | 0.042 | 0.09 (0.08-0.10) | 0.11 (0.10-0.13) | 0.049 | 0.032 |
| Pizza | 0.11 (0.09-0.13) | 0.12 (0.10-0.14) | 0.462 | 0.024 | 0.16 (0.15-0.17) | 0.17 (0.16-0.18) | 0.271 | 0.016 | 0.11 (0.10-0.13) | 0.14 (0.13-0.15) | 0.002 | 0.052 |
| Fried food | 0.40 (0.34-0.46) | 0.44 (0.38-0.50) | 0.438 | 0.025 | 0.51 (0.47-0.54) | 0.53 (0.49-0.56) | 0.334 | 0.014 | 0.43 (0.40-0.47) | 0.54 (0.50-0.58) | <0.001 | 0.067 |

| **Older adults (≥65 years)** | | | | | | | | | | | | |
| --- | --- | --- | --- | --- | --- | --- | --- | --- | --- | --- | --- | --- |
| **Variables** | **Normal**  ***n* = 305** | **LMHF**  ***n* = 305** | **P-value** | **Effect size** | **Normal**  ***n* = 678** | **LMo**  ***n* = 678** | **P-value** | **Effect size** | **Normal**  ***n* = 625** | **HFo**  ***n* = 625** | **P-value** | **Effect size** |
| Carbohydrates (%) | 76.1  (75.1-77.2) | 73.7  (72.7-74.8) | 0.002 | 0.128 | 75.8  (75.1-76.5) | 75.3  (74.6-76.0) | 0.330 | 0.027 | 76.4  (75.7-77.1) | 74.6  (73.8-75.3) | <0.001 | 0.053 |
| Carbohydrates (g) | 300.5  (295.7-305.4) | 290.9  (286.0-295.7) | 0.007 | 0.111 | 303.5  (300.3-306.7) | 302.2  (299.0-305.4) | 0.580 | 0.015 | 302.1  (298.8-305.5) | 295.5  (292.1-298.8) | 0.006 | 0.060 |
| Proteins (%) | 12.7  (12.3-13.1) | 13.6  (13.2-14.0) | 0.004 | 0.119 | 13.0  (12.7-13.2) | 13.0  (12.7-13.3) | 0.790 | 0.007 | 12.7  (12.4-13.0) | 13.2  (12.9-13.5) | 0.013 | 0.042 |
| Proteins (g) | 51.8  (50.0-53.6) | 55.3  (53.5-57.0) | 0.008 | 0.108 | 54.0  (52.8-55.3) | 53.9  (52.7-55.1) | 0.881 | 0.004 | 52.3  (50.9-53.7) | 54.3  (52.9-55.7) | 0.045 | 0.049 |
| Fats (%) | 11.2  (10.4-12.0) | 12.7  (11.9-13.5) | 0.008 | 0.108 | 11.3  (10.8-11.8) | 11.7  (11.2-12.2) | 0.246 | 0.032 | 10.9  (10.4-11.5) | 12.2  (11.7-12.8) | 0.001 | 0.045 |
| Fats (g) | 20.8  (19.2-22.5) | 23.6  (21.9-25.2) | 0.021 | 0.094 | 21.7  (20.6-22.8) | 22.3  (21.2-23.4) | 0.426 | 0.022 | 20.8  (19.7-22.0) | 22.9  (21.8-24.0) | 0.010 | 0.051 |
| Weekly consumption frequency (times per week) of food groups | | | | | | | | | | | | |
| Rice | 19.93 (19.57-20.28) | 19.53 (19.16-19.89) | 0.127 | 0.064 | 19.85 (19.64-20.07) | 19.95 (19.73-20.17) | 0.528 | 0.018 | 19.83 (19.58-20.08) | 19.27 (19.02-19.53) | 0.002 | 0.089 |
| Mixed grain rice | 12.99 (11.99-13.98) | 13.55 (12.54-14.57) | 0.439 | 0.032 | 12.66 (11.96-13.36) | 12.84 (12.13-13.54) | 0.729 | 0.01 | 13.22 (12.54-13.91) | 13.50 (12.81-14.19) | 0.576 | 0.016 |
| Bread and rice cakes | 0.91 (0.75-1.07) | 0.89 (0.73-1.06) | 0.859 | 0.007 | 0.96 (0.84-1.08) | 0.90 (0.78-1.02) | 0.525 | 0.018 | 0.85 (0.72-0.98) | 1.09 (0.96-1.22) | 0.010 | 0.075 |
| Snacks | 0.49 (0.34-0.64) | 0.49 (0.34-0.64) | 0.969 | 0.002 | 0.45 (0.36-0.54) | 0.47 (0.37-0.56) | 0.827 | 0.006 | 0.41 (0.29-0.53) | 0.54 (0.41-0.66) | 0.150 | 0.042 |
| Vegetables | 28.82 (27.26-30.37) | 29.04 (27.45-30.62) | 0.847 | 0.008 | 29.46 (28.42-30.50) | 30.15 (29.10-31.19) | 0.362 | 0.025 | 29.47 (28.42-30.52) | 29.07 (28.01-30.12) | 0.598 | 0.015 |
| Beverages | 0.18 (0.12-0.24) | 0.22 (0.15-0.28) | 0.415 | 0.034 | 0.22 (0.14-0.30) | 0.25 (0.18-0.33) | 0.560 | 0.016 | 0.19 (0.13-0.26) | 0.24 (0.18-0.31) | 0.291 | 0.031 |
| Coffee | 7.18 (6.43-7.94) | 6.33 (5.57-7.10) | 0.126 | 0.064 | 7.35 (6.84-7.86) | 7.19 (6.68-7.70) | 0.662 | 0.012 | 6.36 (5.86-6.86) | 6.68 (6.18-7.18) | 0.384 | 0.025 |
| Alcoholic beverages | 1.24 (0.91-1.57) | 1.02 (0.69-1.36) | 0.378 | 0.037 | 1.63 (1.31-1.96) | 2.19 (1.86-2.52) | 0.019 | 0.066 | 1.17 (0.95-1.39) | 0.86 (0.64-1.07) | 0.046 | 0.058 |
| Ultra-processed food | 0.15 (0.10-0.19) | 0.15 (0.11-0.20) | 0.813 | 0.010 | 0.12 (0.10-0.15) | 0.13 (0.11-0.16) | 0.605 | 0.014 | 0.13 (0.11-0.15) | 0.14 (0.12-0.17) | 0.463 | 0.021 |
| Hamburger | 0.02 (0.00-0.03) | 0.02 (0.01-0.04) | 0.740 | 0.014 | 0.01 (0.00-0.02) | 0.01 (0.00-0.02) | 0.953 | 0.002 | 0.01 (0.01-0.02) | 0.01 (0.00-0.01) | 0.302 | 0.030 |
| Pizza | 0.02 (0.01-0.03) | 0.03 (0.01-0.04) | 0.397 | 0.035 | 0.02 (0.01-0.02) | 0.01 (0.00-0.02) | 0.068 | 0.051 | 0.02 (0.01-0.03) | 0.02 (0.01-0.03) | 0.796 | 0.007 |
| Fried food | 0.11 (0.08-0.14) | 0.11 (0.07-0.14) | 0.870 | 0.007 | 0.10 (0.08-0.12) | 0.11 (0.09-0.13) | 0.228 | 0.034 | 0.10 (0.08-0.11) | 0.11 (0.10-0.13) | 0.169 | 0.040 |

LMHF, low muscle mass with high body fat; HFo, high body fat only, but not low muscle mass; LMo, low muscle mass only, but not high body fat. Ultra-processed food includes hamburger, pizza, and fried food, which were also analyzed separately. The values are presented as ‘estimated mean (95% confidence interval)’ for each variable. *P*-values are calculated using the analysis of covariance after adjusting for total calories.
